# Analysis of the Effect of Protective Properties of Concretes with Similar Composition on the Corrosion Rate of Reinforcing Steel Induced by Chloride Ions

**DOI:** 10.3390/ma16103889

**Published:** 2023-05-22

**Authors:** Zofia Szweda, Justyna Kuziak, Liwia Sozańska-Jędrasik, Dominik Czachura

**Affiliations:** 1 Department of Building Structures, Faculty of Civil Engineering, Silesian University of Technology, 44-100 Gliwice, Poland; 2 Institute of Building Engineering, Department of Building Materials Engineering, Faculty of Civil Engineering, Warsaw University of Technology, 00-637 Warsaw, Poland; justyna.kuziak@pw.edu.pl; 3 Łukasiewicz Research Network–Upper Silesian Institute of Technology, Centre of Welding, 44-100 Gliwice, Poland; liwia.sozanska-jedrasikl@imz.lukasiewicz.gov.pl; 4 Smart Solutions, Measurement Center 3D, 03-046 Warsaw, Poland; dominik.czachura@smart-solutions.pl

**Keywords:** composition of concrete, corrosion rate, impedance spectroscopy, linear polarization, microdiffraction, porosity of concretes

## Abstract

This study presents a comparison of the protective properties of three concretes of similar composition on the effect of chloride ions. To determine these properties, the values of the diffusion and migration coefficients of chloride ions in concrete were determined using both standard methods and the thermodynamic ion migration model. We tested a comprehensive method for checking the protective properties of concrete against chlorides. This method can not only be used in various concretes, even those with only small differences in composition, but also in concretes with various types of admixtures and additives, such as PVA fibers. The research was carried out to address the needs of a manufacturer of prefabricated concrete foundations. The aim was to find a cheap and effective method of sealing the concrete produced by the manufacturer in order to carry out projects in coastal areas. Earlier diffusion studies showed good performance when replacing ordinary CEM I cement with metallurgical cement. The corrosion rates of the reinforcing steel in these concretes were also compared using the following electrochemical methods: linear polarization and impedance spectroscopy. The porosities of these concretes, determined using X-ray computed tomography for pore-related characterization, were also compared. Changes in the phase composition of corrosion products occurring in the steel–concrete contact zone were compared using scanning electron microscopy with a micro-area chemical analysis capability, in addition to X-ray microdiffraction, to study the microstructure changes. Concrete with CEM III cement was the most resistant to chloride ingress and therefore provided the longest period of protection against chloride-initiated corrosion. The least resistant was concrete with CEM I, for which, after two 7-day cycles of chloride migration in the electric field, steel corrosion started. The additional use of a sealing admixture can cause a local increase in the volume of pores in the concrete, and at the same time, a local weakening of the concrete structure. Concrete with CEM I was characterized as having the highest porosity at 140.537 pores, whereas concrete with CEM III (characterized by lower porosity) had 123.015 pores. Concrete with sealing admixture, with the same open porosity, had the highest number of pores, at 174.880. According to the findings of this study, and using a computed tomography method, concrete with CEM III showed the most uniform distribution of pores of different volumes, and had the lowest total number of pores.

## 1. Introduction

Depending on their location and function, building structures are exposed to many harmful factors. The most common causes of reinforcement corrosion and concrete damage are carbonation and chloride penetration [1]. The faster the carbon dioxide or chlorides penetrate the concrete, the faster the passive layer on the rebar is destroyed and the corrosion process begins [2]. A very important issue is the corrosion of the reinforcement in foundations that are exposed to the harmful effects of chloride ions present in groundwater, especially in coastal environments, but also in industrial areas [3]. The use of traditional protective measures, consisting of coating the surface of concrete with various insulating layers, is expensive and time-consuming. At the same time, often after a short period of use of the building, these layers decompose in an aggressive and humid ground environment, ceasing to fulfill their protective role. In addition, the products resulting from the decomposition of these coatings are a source of environmental contamination [4]. Therefore, it is important to use such a quality of concrete that, with a properly selected thickness of concrete lagging, can provide a sufficiently good protective barrier to delay the penetration of chloride ions, thus ensuring the safe use of the building structure. The rate of penetration of chloride ions into concrete is characterized by the value of the diffusion coefficient. Currently, there are many methods for determining the value of this coefficient. The first group of methods consists of long-term diffusion tests [5], while another group of methods is based on electric field-accelerated migration tests [6]. Unfortunately, the values of coefficients obtained on the basis of tests conducted according to different standards and methods will ultimately differ from each other [7]. In one study [8], a number of various methods for determining diffusion and migration coefficients that can be carried out on different types of concrete were analyzed, and compared against the method of determining the diffusion coefficient conducted in accordance with the thermodynamic migration model [9]. This analysis showed that this coefficient accurately describes the natural process of chloride ion penetration into concrete.

A factor affecting the rate of chloride ion penetration into concrete is undoubtedly its porosity. However, the relationship between pore diameter and chloride diffusivity is reported to be linear by some researchers, such as Moon et al. [10] and Schutter [11], while Sherman et al. [12] found that the correlation between chloride ion migration coefficient and saturation was very low. Similarly, in one study [13], it has been shown that there is no clear correlation between chloride migration rate and pore size. There is also a hypothesis that in cementitious materials there are both ink-bottle pore and closed pore types, through which the penetration of chloride ions is strongly limited. However, these are detectable using a modified mercury porosimetry method [14,15], in addition to a gravimetric method. It was proven that the dominant factor affecting the value of the diffusion coefficient can often be the calcium aluminate content of the cement contained in the concrete [16]. It is difficult to find a correlation between porosity and diffusion coefficient values in such a complex material as concrete due to the fact that both the methods for determining porosity and the methods of determining the diffusion coefficient are dependent on many variables, and it thus is difficult to make these experiments repeatable. Therefore, a comprehensive analysis of the properties of concrete is necessary to assess its protective properties.

In recent years, the X-ray computed microtomography method (XRD) has been used to determine the porosity of concrete. This method, although promising, is not without some disadvantages. The main problem is the relationship between sample size and spatial resolution. In order to obtain a sufficiently high spatial resolution, it is important to use a sufficiently small sample. However, such a small section of the tested material may not represent the properties of the whole object [17,18]. Despite this, the method is being used with increasing frequency to determine both the porosity of cementitious materials and to visualize the corrosion processes occurring in reinforced concrete materials [19,20,21].

In addition to evaluating the diffusion and porosity properties, it is necessary to assess the rate of development in corrosion processes against the reinforcement in concrete. To evaluate the rate of corrosion development, nondestructive polarization methods are used: the Linear Polarization Resistance (LPR) method [22], and electrochemical impedance spectroscopy (EIS) [23]. Since the diffusion processes are long-lasting, the action of an electric field was used to accelerate the penetration of chloride ions into the concrete, similar to the work [24,25], while the corrosion processes occurred naturally.

The type of corrosion products of reinforcement (i.e., the type of oxides formed) depends on the environment in which the corroding elements are located. The initial stages of atmospheric corrosion of carbon steel in both rural and urban atmospheres leads to the formation of lepidocrocite (γ-FeOOH) and goethite (α-FeOOH), which are the main phases present in corrosion products, regardless of the environment. In chloride-containing coastal environments, akaganeite (β-FeO(OH,Cl)), magnetite (Fe_3_O_4_), and siderite (FeCO_3_) are common. However, under simulated coastal environments, ferrihydrite (Fe(OH)_3_) is also found [26,27]. Corrosion products accumulating on the surface of reinforcing steel in concrete, while increasing their volume, cause tensile stresses that induce cracking in the concrete lagging. Depending on the type of corrosion products, different levels of increased volume are observed. The largest, almost a sixfold increase, is observed when the corrosion products consist of 100% Fe(OH)_3_ ⸱3H_2_O. In contrast, Fe(OH)_2_ and Fe(OH)_3_ hydroxides increase in volume by 2.17 and 1.76 times more than noncorroded steel, respectively. Determining the type of corrosion products is important in the process of modeling the cracking of concrete lagging [28].

The purpose of this study was to test the effectiveness of Improving the protective properties of the plain concrete used in the production of precast foundations. Both the modification of the concrete mixture by replacing Portland cement CEM I 42.5 R with metallurgical cement CEM III/A 42.5 N-LH/HSR/NA, in addition to using a sealing admixture in another mixture, were proposed. A number of tests were carried out to evaluate the efficacy of the proposed modifications on the mechanical and protective properties of the steel reinforcements of building foundations against corrosion processes. The novelty in the work is in using a comprehensive method for determining the resistance of concrete to the action of chloride ions. This method can be used in a variety of concretes, not only in those with only slight differences in composition, but also in concretes with various types of admixtures and additives, such as PVA fibers or the addition of other postindustrial waste. In order to determine the protective properties of concrete, the value of chloride ion diffusion coefficients in concrete was determined using the standard methods according to: ASTM1220, NT BUILD 443, NT BUILD 449, and ASTM1556, as well as using the thermodynamic ion migration model. The corrosion rate of reinforcing steel in these concretes was also compared using electrochemical methods: linear polarization LPR and impedance spectroscopy EIS. The porosities of these concretes (determined using the weight method, in addition to using X-ray computed tomography for pore-related characterization) were also compared. Using scanning electron microscopy (SEM) with microarea chemical analysis capabilities, as well as X-ray microdiffraction (XRD) to study microstructural changes, changes in the phase composition of corrosion products occurring in the steel–concrete contact zone were compared. It turns out that a slight modification of the composition of the concrete used can result in a huge improvement in its protective properties, ensuring the safety and durability of the reinforced concrete structures made from it.

## 2. Materials

### 2.1. Type of Material Tested

The tests were performed on three types of concrete mix. All of the concretes tested were designed to be ordinary concretes (used for precast foundation elements). Three types of natural rounded aggregate (from Lafarge Basalt Mine in Lubień, compliant with the standard PN-EN 12620 [29]) were utilized for mix creation: sand, 0 ÷ 2 mm (722 kg/m^3^); gravel, 2 ÷ 8 mm (512 kg/m^3^); and gravel, 8÷16 mm (681 kg/m^3^). The first tested concrete (B1) contained CEM I 42.5 R cement in its composition. The next concrete (B2) differed from the first only in the use of CEM III/A 42.5 N-LH/HSR/NA cement. The last concrete (B3) was further modified using a refining admixture in the amount recommended by the manufacturer (0.5% of the weight of the cement). The detailed compositions and properties of mixes are presented in Table 1.

### 2.2. Type of Samples Used in the Tests

The compressive strength of the concrete was determined using cubic samples possessing 150 mm sides, which were stored and made in accordance with PN-EN12350-3:2011 [30].

## 3. Test Method

Next, a series of tests was conducted to determine the protective properties of the tested concretes against the effects of corrosion on the concrete’s reinforcements by chloride ions. In order to perform these tests, 12 samples of each type of concrete were prepared.

To determine the depth of penetration by the pressurized water, cubic samples with 150 mm sides were used. In order to perform these tests, 3 samples of each type of concrete were prepared.

To determine the open porosity of hardened concrete, 1 sample for every type of concrete (each with a volume of about 10 cm^3^) was used.

**Figure 1:**
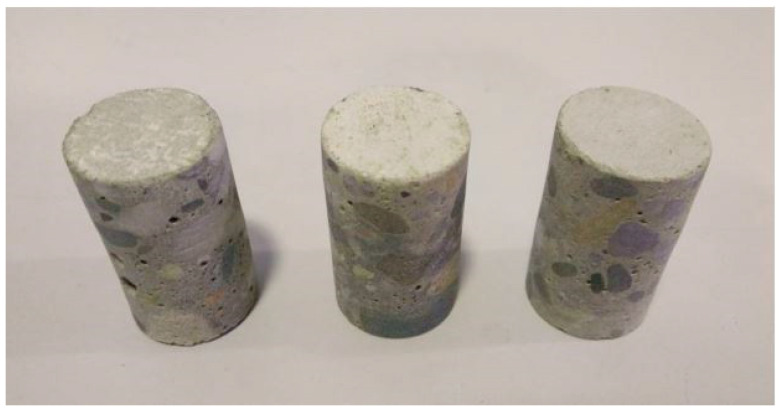
Concrete cores prepared for testing using X-ray computed microtomography.

The X-ray computed microtomography tests used concrete cores with a diameter of about 18 mm and a height of 50 mm, cut with a diamond core drill from cylindrical specimens with a diameter of 100 mm and a height of 50 mm (Figure 1).

For examination of the rate of penetration of chloride ions into concrete, concrete disks that were 50 mm thick and 100 mm in diameter were used. In order to perform these tests, 21 samples for each type of concrete were prepared.

**Figure 2:**
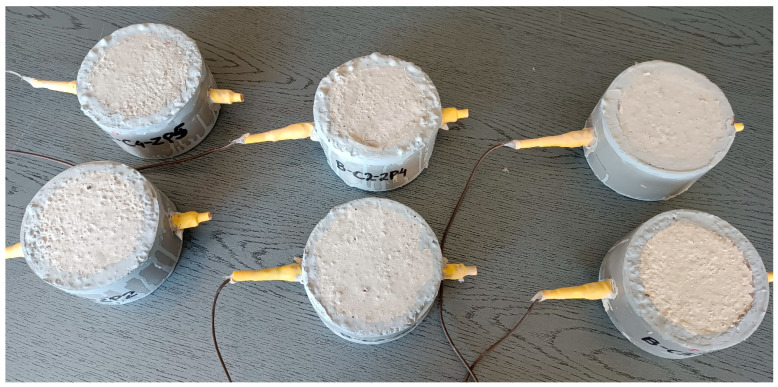
Concrete samples prepared for testing using non-destructive polarization tests (LPR and EIS).

The non-destructive polarization tests (LPR and EIS) were carried out each time on 2 test pieces in the shape of cylinders that were 100 mm in diameter and 60 mm high, made of each of the three types of concrete. A reinforcing bar was placed in each cylinder. A reinforcement cover 20 mm in thickness was used (Figure 2).

**Figure 3:**
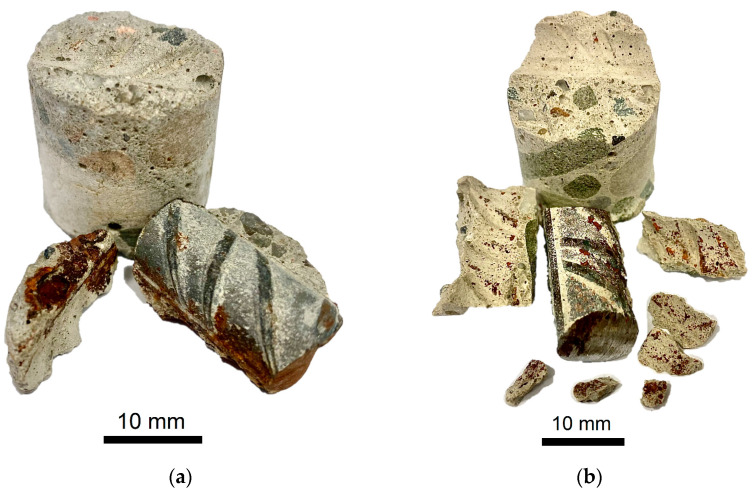
Examples of two smaller cylindrical test tubes that were cut from larger cylindrical test tubes previously subjected to chloride ion migration and corrosion measurements: (**a**) concrete sample B1; (**b**) concrete sample B2.

The research for phase identification and quantitative analysis (using the Rietveld method) of corrosion products was conducted on three rebar specimens in concrete lagging made of 3 types of concrete, following simulations of the deterioration process under the formation of corrosion hazards caused by the presence of chloride ions. Figure 3 shows two smaller cylindrical test specimens made of B1 and B2 concrete; these had been cut out of the larger cylindrical test specimens tested, as outlined in Section 3.6, using a diamond-core drill so as to minimize rebar fragments. The samples disintegrated during cutting.

### 3.1. Compressive Strength Test after 1, 2, 7, and 28 Days of Maturation

The test was conducted after 1, 2, 7, and 28 days of concrete curing, which had been performed with the use of machine for testing the compressive strength of concrete. The results are shown in Table A1, Table A2 and Table A3 of the Appendix A.

### 3.2. Determination of the Depth of Penetration of Pressurized Water

Testing the depth of water penetration under pressure was performed according to EN 12390-8, Testing of concrete: Part 8: Depth of water penetration under pressure [31]. A water pressure of 0.5 MPa was maintained for 72 h. After this time, the sample was split, to determine the moisture level.

### 3.3. Tests Open Porosity of Hardened Concrete

Open porosity was defined as the ratio of the differences between $m_{SD}$ (the weight of the specimens completely saturated with water) and $m_{OD}$ (the weight of the specimens dried at 60 °C); in both cases, the weight of the specimens was checked daily until the change in the weight of the specimens was less than 0.1%) against the differences between $m_{SD}$ (the weight of the specimens completely saturated with water) and $m_{FD}$ (the floating weight of the specimens with the use of hydrostatic balance), calculated according to the following equation

(1) $$\varepsilon =\frac{m_{SD}-m_{OD}}{m_{SD}-m_{FD}}\cdot 100\%$$

### 3.4. Use of X-Ray Computed Microtomography to Determine the Porosity of Concrete

A Nikon Corporation Industrial Metrology Business Unit, Tokyo, Japan XT H 225 ST industrial microtomograph equipped with a reflection lamp was used for the tomographic study; at the maximum approximation of small specimens, it proposed a resolution of 3 µ. The optimal resolution used in this case was 10 µ, which made it possible to obtain reliable images of the scanned sample. The resulting images were then reconstructed using Nikon’s CTPro 3D 6th generation software. After reconstruction, data preparation and all analyses were carried out using the appropriate tools available in VGStudio MAX 3.1 software. Prior to defectoscopy, an area 13 mm in diameter and 15 mm in height, slightly smaller than the scanned area, was taken from each scanned core. Clipping was necessary due to artifacts and imperfections in the tomographic image created during scanning. The data prepared in this way was analyzed for porosity.

### 3.5. Examination of the Rate of Penetration of Chloride Ions into Concrete

#### 3.5.1. Testing the Diffusion of Chloride Ions through a Concrete Sample, in Accordance with the Norwegian Standard NT BUILD443 [32] and the American Standard ASTM1556 [33]

The test was conducted in accordance with the standards NT BUILD 443 [32] and ASTM C 1556-03 [33]. The test was carried out on three specimens per type of concrete in each case. The exact test method is described in the paper [8].

The value of the *D* coefficient is determined by adjusting the chloride concentration graph. This graph is obtained by calculating the distribution of chloride ion concentration expressed to the cement mass according to the solution of the diffusion equation, with the concentrations of these ions determined in the test:

(2) $$C_{cal}^{1}\left(x,t\right)=C_{0,cal}^{1}\left(1-erf\frac{x}{2\sqrt{Dt}}\right)$$

To determine the best fit calculation of the diffusion coefficient complying with the results of the experiment, the mean squared error was calculated based on the following formula:

(3) $$s=\sqrt{\frac{\sum_{i=1}^{n}{\left[c_{cal}\left(x,t\right)-c\left(x,t\right)\right]}^{2}}{n-1}}$$

where *c(x,t)* is the chloride ion concentration that was measured during the test at a distance *x* (mm) from the edge of the element (%), *t* is time (s), and *n* is the number of concrete layers in which the chloride concentration was determined. The determined values of the diffusion coefficient are presented in Section 4.5.

#### 3.5.2. Testing the Permeability of Chloride Ions through a Concrete Sample, in Accordance with American Standards AASHTO T 277 [34] and ASTM C1202-97 [35]

The study was conducted in accordance with the procedure described in standards AASHTO T 277 [34] and ASTM C1202-97 [35], each time being performed on three samples made of each concrete type.

Based on the determined charge, the value of the diffusion coefficient was also calculated using the Nernst–Einstein equation

(4) $$D_{NE}=\frac{RT}{z^{2}F^{2}}\frac{t_{i}}{C_{i}\gamma_{i}\rho_{BR}},\;\;\;\rho_{BR}=\frac{100}{\sigma },\;\;\;\sigma =\frac{QL}{VtA}$$

where $D_{NE}$ is the diffusion coefficient (m^2^/s), R is the universal gas constant (J/Kmol), T is absolute temperature (K), z is the valence of ions (-), F is the Faraday constant (C/mol), $t_{i}$ is 1 transport number of chloride ions (-), $\gamma_{i}$ is 1 activity coefficient of chloride ions (-), $C_{i}$ is the concentration of chloride ions (mol/m^3^), $\rho_{BR}$ is volumetric resistivity (Ωm), σ is conductivity (Ωm^−1^), *L* is sample thickness (m), *V* is electrical potential (V), *A* is the cross-sectional area of a sample (m^2^), and *t* is time (s).

#### 3.5.3. Testing the Permeability of Chloride Ions through a Concrete Sample, in Accordance with the Norwegian Standard NT BUILD492 [36]

The tests were carried out in accordance with the procedure described in NT BUILD492 [36], being performed each time on three samples per concrete type. The exact method of testing is described in the paper [8].

In accordance with the standard NT BUILD492 [36], the migration coefficient was calculated on the basis of the penetration depth, which was determined with the colorimetric method using a 0.1 M silver nitrate AgNO_3_ solution, according to the formula:

(5) $$\alpha =2\sqrt{\frac{RTL}{zFU}}erf^{-1}\left(1-\frac{2c_{d}}{c_{0}}\right),\;\;\;\;\;\;\;\;\;\;\;D_{T}=\frac{RTL}{zFU}\frac{x_{d}-\alpha \sqrt{x_{d}}}{t_{d}}$$

where $c_{0}$ is the concentration of chlorides in the source chamber and the concrete, respectively, to the depth $x_{d}$; $t_{d}$ is test duration (hours); L is element thickness, (mm); U is the value of the applied voltage; $c_{d}$ is the chloride concentration at which the color changes (0.07 M for OPC concrete); $c_{0}$ is the chloride concentration in the cathode solution (2 M); and $erf^{-1}$ is the inverse of the error function.

The depth of chloride penetration was determined in one of the three test samples made from each concrete.

In turn, the other two samples were used to determine the distribution of chloride ion concentration in concrete using the method described in the papers [8,9,37]. In this method, a device called a Profile Grinding Kit from Germann Instruments was used to obtain concrete dust from ten layers, each 2 mm thick. Then, after obtaining solutions by modeling the pore liquid, chloride ion steepness was measured with a CX-701 multimeter from Elmetron, using an ion-selective electrode. The value of the diffusion coefficient was determined using Equations (2) and (3). The determined values of the diffusion coefficient are presented in Section 4.5.

#### 3.5.4. Migration Studies and Determination of Diffusion Coefficient Values Based on the Thermodynamic Migration Model

Migration tests were conducted using the method described in the papers [8,9], and [37]. Six cylindrical specimens, 100 mm in diameter and 50 mm in height, were made from each concrete type, and examined. Chloride migration tests were conducted at two time points: t_1_ = 24 and t_2_ = 48 h. At the end of migration, the level of chloride ion concentration in the water extracts obtained from stratified stripped concrete was determined using a Profile Grinding Kid device with both a diamond drill and an attachment that allowed the extraction of 2 mm thick concrete layers, to a depth of 20 mm.

Based on measurements of the mass density distribution $\rho_{1}$ of chloride ions migrating in concrete under the influence of an electric field, the authoritative value of the diffusion coefficient was determined using the relationship [8,9,37]

(6) $$D^{1}=\frac{\bar{j^{1}}\left(a\right)a\Delta t}{\frac{z^{1}FUg}{RTh}\left[\bar{\rho_{1}^{1}}+\bar{\rho_{2}^{1}}+\ldots +\bar{\rho_{n}^{1}}\right]\Delta t-B},\;B≅\omega \frac{z^{1}FUg}{RTh}\left(\bar{\rho_{1}^{1}}+\bar{\rho_{2}^{1}}+\ldots +\bar{\rho_{n}^{1}}\right)\Delta t.$$

In this expression, $\bar{j^{1}}\left(a\right)$ is the value of the mass flow of chloride ions passing through the plane situated at “a” distance x=a; $\bar{\rho_{1}^{1}}$, $\bar{\rho_{2}^{1}}$, and $\bar{\rho_{n}^{1}}$ are the averaged mass densities of ion Cl^−^ at the midpoints of consecutive intervals $\left(0,g\right)$, $\left(g,2g\right)$, …,$\left[\left(n-1\right)g,a\right]$ in time Δt. The first component of the denominator defines the stationary part of the chloride ion flow, while the second component (B) defines the nonstationary part (in this study, the value *B* = 0). Furthermore, *z*^1^ is the ion valence, *R* = 8.317 J/mol·K is the universal gas constant, *F* = 96 487 C/mol is the Faraday constant, *U* is the voltage between the electrodes, and *h* is the specimen height. The determined values of the diffusion coefficient are presented in Section 4.5.

#### 3.5.5. Diffusion Tests

Diffusion tests were carried out using the method described in the papers [8,9,37]. Six cylindrical specimens for each concrete, each 100 mm in diameter and 50 mm in height, were examined each time. Chloride migration tests were conducted at two time points: t_3_ = 30 and t_4_ = 60 days. After diffusion, the value of chloride ion concentration in the water extracts was determined in the same way as after migration tests.

### 3.6. Measurements of Linear Polarization Resistance LPR

Polarization studies of the corrosion rate in the reinforcements using a polarization resistance measurement method (LPR) were carried out using a potentiostat (Gamry Reference 600, made by Gamry Instruments, Warminster, United States of America), coupled with a computer set. The measurement was recorded in a three-electrode system in which the working electrode was the sample reinforcement, the reference electrode was a silver chloride electrode, and the counterelectrode was a stainless-steel circle with a diameter close to that of the concrete samples. In order to accelerate the very long timescale for adequate chloride diffusion in the concrete, accelerated electromigration of chloride ions was used, similar to the works [24,25], while the corrosion processes induced due to the threshold chloride content of concrete occurred naturally. The corrosion current $i_{corr}$ could be calculated via polarization resistance $R_{p}$ obtained using an LPR measurement, according to the Stern–Geary equation

(7) $$R_{p}={\left.\frac{dE}{di}\right|}_{i\overset{}{\to }0,\;E\overset{}{\to }E_{corr}},i_{corr}=\frac{b_{a}b_{c}}{2.303R_{p}\left(b_{a}+b_{c}\right)},$$

where $b_{a}$ is constant of anodic reactions, and $b_{c}$ is constant of cathodic reactions.

The corrosion current density clearly determines the corrosion intensity of steel, and this is because, according to Faraday’s law, the mass of losses ($m\left(\mathrm{mg}\right))$ is proportional to the flowing current ($I_{corr}($μA/cm^2^))

(8) $$\Delta m=kI_{corr}t,I_{corr}=\frac{i_{corr}}{A},$$

where *k* is electrochemical equivalent, and *t* is time. The above relationship shows the correlation of the corrosion current density, with the linear corrosion rate ($V_{r}$ (mm/year) expressed as follows:

(9) $$V_{r}=0.0116\;i_{corr}$$

Corrosion rate $(V_{r}$ (mm/year) is determined from the average cross-section loss around the bar circumference, in mm, per 1 operational year of the structure. The detailed results from the analysis with the calculated densities for corrosion current are shown in Table A4, Table A5 and Table A6 of the Appendix B.

### 3.7. EIS Measurements for Steel in Concrete

Measurements of EIS spectra for steel in concrete were carried out before the LPR measurements in the same measurement set-up, and on the same samples as the LPR measurements described in Section 3.6. EIS spectra were recorded at the corrosion potential in the frequency range of 100 kHz to 50 mHz. The amplitude of the perturbation signal was 10 mV, and 10 points per decade of frequency were recorded.

**Figure 4:**
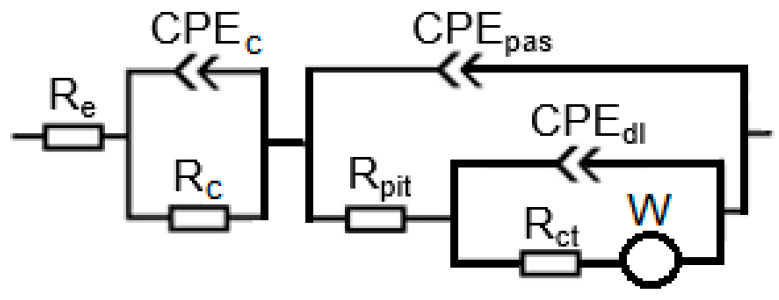
Equivalent circuit for steel in concrete (R_e_ is the electrolyte resistance; R_c_ is concrete resistance; CPE_c_ is the constant phase element describing concrete cover; R_pit_ is the ohmic resistance in pits or defects of passive layer; CPE_pas_ is the constant phase element for passive surface; R_ct_ is the charge transfer resistance; CPE_dl_ is the constant phase element describing double layer on the steel; and W is the Warburg (diffusional) impedance.

The impedance spectra obtained were analyzed using the electrical equivalent circuit shown in Figure 4. The part of the spectra recorded at high frequencies describes the properties of the concrete and corresponds to the left part of the electrical equivalent circuit, which contained the following elements: the electrolyte resistance (R_e_), concrete resistance (R_c_), and constant phase element describing concrete cover (CPE_c_). The impedance spectra at medium and high frequencies characterizes steel, and corresponds to the right-hand part of the electrical equivalent circuit, which contains the ohmic resistance in pits or defects of the passive layer (R_pit_), the constant phase element for passive surface (CPE_pas_), the charge transfer resistance (R_ct_), constant phase element describing double layer on the steel (CPE_dl_), and the Warburg (diffusional) impedance (W).

### 3.8. Phase Identification and Quantitative Analysis of Corrosion Products, Using the Rietveld Method 

Measurements were performed with an Empyrean X-ray diffractometer acquired from PANalytical, using filtered iron radiation in a pixel detector configuration. Identification of phase composition was carried out in accordance with the M1-RTG accredited procedure, entitled “*Phase Identification*”, in addition to the International Centre for Diffraction Data’s PDF-4+ database. Quantitative analysis was performed using the Rietveld method, in accordance with the accredited procedure M2-RTG, entitled “*Quantitative Phase Analysis*.” Observations were carried out using a JSM 7200F scanning electron microscope from JOEL, with a BSE backscattered electron detector and an EDS detector, to perform microarea chemical analysis.

## 4. Results

### 4.1. Compressive Strength Test Results after 1, 2, 7, and 28 Days of Maturation

**Figure 5:**
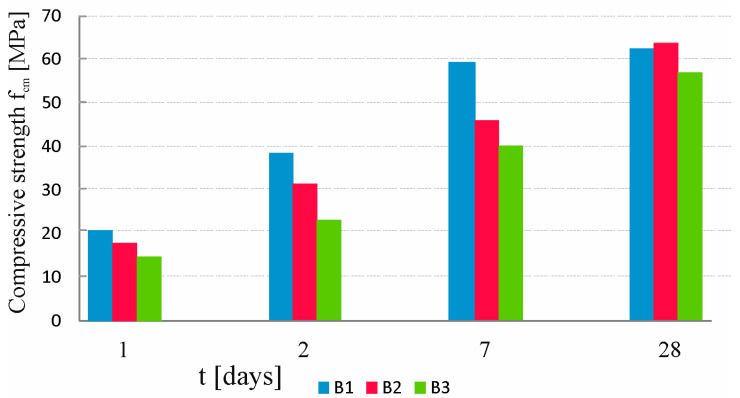
Compressive strength of concretes B1, B2, and B3 after 1, 2, 7, and 28 days of maturation.

Figure 5 shows the compressive strength comparison of the tested concretes.

Changing the cement from Portland cement (for B1 concrete) to metallurgical cement (for B2 and B3 concretes) caused a reduction in compressive strength in the first 7 days of maturation. This reduction in compressive strength could also have been caused by the sealer admixture (B3 concrete). After the 28th day of maturation, the strength of concrete with metallurgical cement was the highest, and a clear upward trend was also visible. The properties of blast furnace slag, which is a component of metallurgical cement, allow us to hypothesize that the strength of B2 and B3 concretes will be higher than that of B1 concrete after a longer maturation time.

### 4.2. Results of Pressurized Water Penetration Depth Tests

Table 2 summarizes the depth of penetration of water under pressure, as determined using the method outlined in the standard PN-EN 12390-8 [31], in three samples made from each concrete.

**Figure 6:**
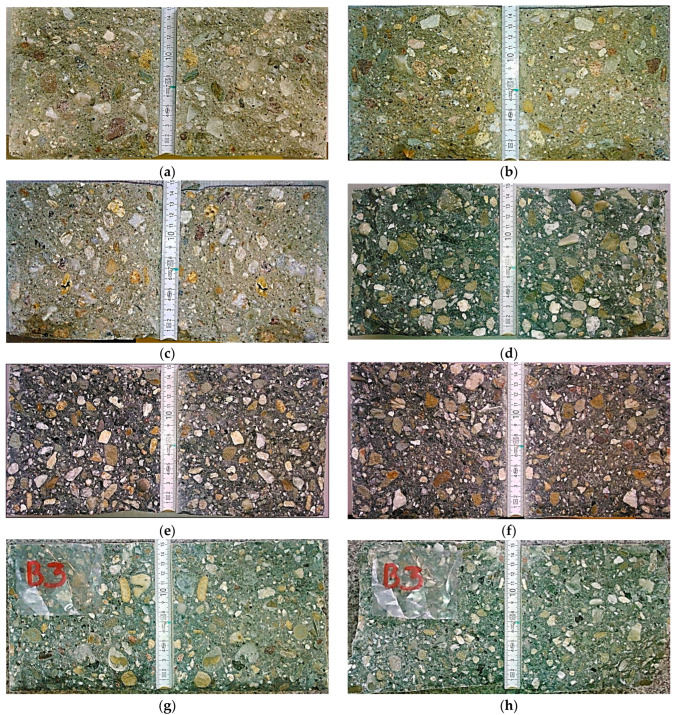

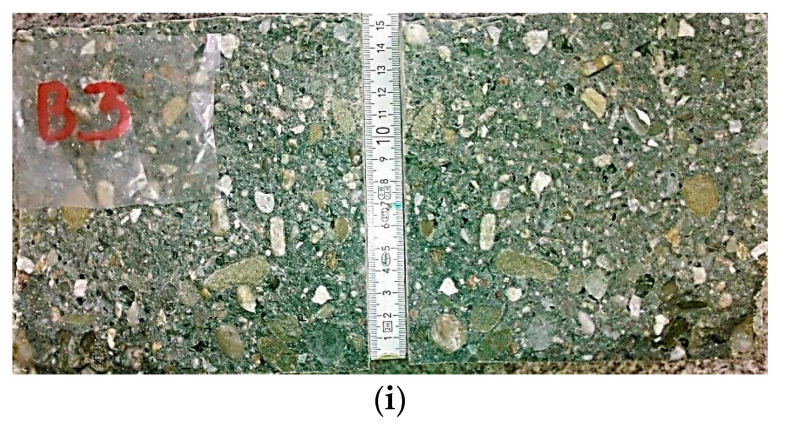
Cross-sections of the test samples obtained after splitting them immediately after moistening with water at a pressure of 0.5 MPa maintained for 72 h: (**a**) sample B1.1; (**b**) sample B1.2; (**c**) sample B1.3; (**d**) sample B2.1; (**e**) sample B2.2; (**f**) sample B2.3; (**g**) sample B3.1; (**h**) sample B3.2; and (**i**) sample B3.3.

Figure 6 shows cross-sections of the test samples, cut after the pressure water penetration test.

The use of metallurgical cement significantly reduced the depth of pressurized water penetration. However, the addition of a sealing admixture did not significantly improve the achieved effect in B3 concrete. It should be noted that the penetration of water deep into each sample was of a local nature. The magnitude of penetration forming about 20 mm was small and appropriate for concretes with a good quality of cement matrix.

### 4.3. Results of Testing the Open Porosity of Hardened Concrete

Table 3 presents the values of the weight of the sample determined using the gravimetric method with a hydrostatic balance, and the porosity calculated according to the formula (1).

As can be seen from the results, the tested concretes did not differ substantially in terms of open porosity as determined using the gravimetric method. Concrete B3 (with sealing admixture) had slightly better properties than the other concretes.

### 4.4. Results of Porosity Analysis of Concrete Cores using X-ray Computed Microtomography

**Figure 7:**
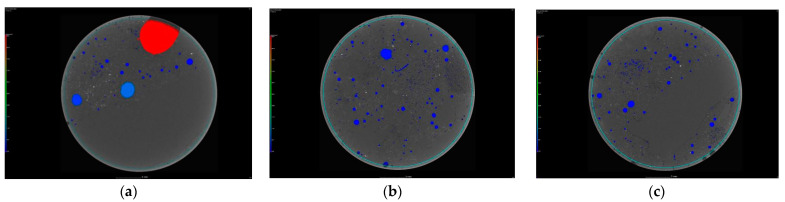

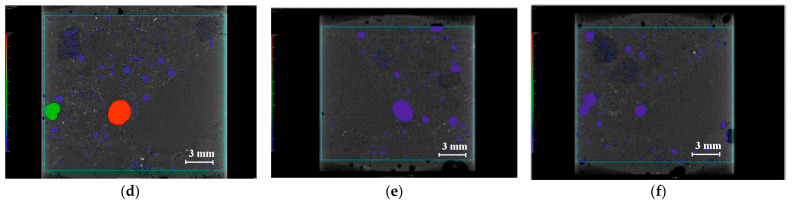
Selected cross sections taken during the tomographic examination, together with the image analysis performed in VGStudio MAX 3.1 software: cross section of the cylinder (**a**) concrete B1, (**b**) concrete B2, and (**c**) concrete B3; longitudinal cross-section of the cylinder (**d**) concrete B1, (**e**) concrete B2, and (**f**) concrete B3.

Figure 7 shows cross sections made in B1, B2, and B3 concrete, subjected to image analysis in VGStudio MAX 3.1 software.

These were based on tomographic studies of a smaller cylindrical sample, measuring 18 mm in diameter and 21 mm in height.

On the basis of the analysis performed, the porosity of the concretes was calculated, expressed as the percentage of the sum of all air voids (imaged using the tomograph) against the volume of the fragment of the cylindrical sample with a diameter of 18 mm and a height of 21 mm, amounting to p_1_ = 14%, p_2_ = 6%, and p_3_ = 6% for concretes B1, B2, and B3, respectively. According to these tomograph tests, it can be said that the replacement of the ordinary cement (CEM I) with metallurgical cement (CEM III) resulted in a reduction in the porosity of the concrete by almost half. On the other hand, the use of a sealing agent had practically no effect on the porosity.

**Figure 8:**
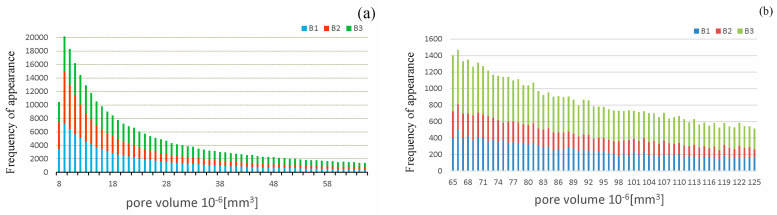

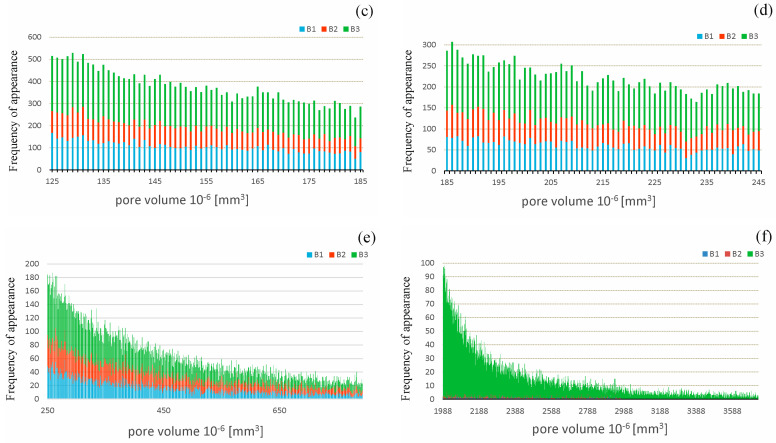
Frequency of pores of a given volume occurring in analyzed concrete samples B1, B2, B3: (**a**) volume range (8–64) × 10^−6^ mm^3^; (**b**) volume range (65–124) × 10^−6^ mm^3^; (**c**) volume range (125–184) × 10^−6^ mm^3^; (**d**) volume range (185–249) × 10^−6^ mm^3^; (**e**) volume range (250–1987) × 10^−6^ mm^3^; (**f**) volume range (1987–3788) × 10^−6^ mm^3^.

Figure 8 shows graphs of the frequency of pores of a given volume occurring in the analyzed concrete samples. Concrete B1 was characterized by the highest porosity, and had 140.537 pores; concrete B2, characterized by having half the porosity of B1, had 123.015 pores, while concrete B3, with the same porosity as B2, had the highest number of pores, at 174.880. Considering the above graphs, it can be concluded that concrete B3 has both the most pores and the highest volume (Figure 8f). Overall, B2 concrete is characterized by the smallest number of pores. Concrete B1 had 1.1 times more pores than concrete B2, while concrete B3 had 1.4 times as many pores. In summary, it can be seen that B2, while having a low porosity compared to concrete B1, simultaneously had fewer pores with a large volume compared to concrete B3.

### 4.5. Determination of the Rate of Penetration of Chloride Ions into Water-Saturated Concretes

All three concretes were tested using both standard methods and the thermodynamic method. A detailed discussion of the methods, including a comparison with the results obtained in different concrete mixtures, are presented in the paper [8]. Table 4 summarizes the diffusion and migration coefficient values determined using various methods. The table includes the values of the diffusion coefficient $D_{NE}$ determined from the charge measured during the migration test, conducted according to the rules of the standard ASTM C1202-997 [35] after applying the Nernst–Einstein Equation (4). Another coefficient specified in the standard ($D_{T}$) was calculated on the basis of migration tests, in accordance with the rules contained in the standard NT BUILD 492 [36], based on the depth of penetration of chloride ions, determined with the colorimetric method using the Equation (2). Subsequent values of the migration rate $D_{migr}^{1}$ were determined by fitting the concentration curve that had been plotted according to Equation (2) to the results obtained in a migration test conducted in accordance with the standard NT BUILD 492 [36]. On the basis of this coefficient, using Equation (2), which in a simplified manner takes into account a certain multiplier linking the diffusion flow to the migration of chloride ions, the value of the diffusion coefficient $D_{dif\;}^{1}$ was determined. Another diffusion coefficient (D) was determined from diffusion tests conducted in accordance with the standard NT BUILD 443 [32]; this was determined using Equation (2) based on the lowest value of the mean–square error (3). The next two values of the diffusion coefficient ($D^{t1}$ and $D^{t2}$) were determined by fitting a concentration curve that had been determined numerically from Equations (4) and (2) based on diffusion tests. Another value of the diffusion coefficient ($D^{1}$) was determined on the basis of both migration studies and Equation (6), which was the solution of the thermodynamic migration model.

According to an analysis of various methods of determining the diffusion coefficient in different types of concrete, each of which having been carried out in the paper [8], the value of diffusion coefficient that best represents the natural diffusion process was the coefficient $D^{1}$, determined from the thermodynamic migration model. The value of this coefficient was used to predict the durability time of a structure made of one of the three concretes considered.

**Figure 9:**
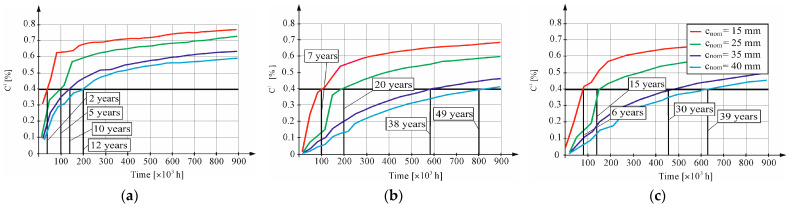
Time₋dependent change in chloride concentration at the surface of the reinforcement at different lagging thicknesses (15, 25, 35, and 40 mm): (**a**) concrete B1, (**b**) concrete B2, and (**c**) concrete B3.

It was assumed that the threat of corrosion of the reinforcing bars causes, according to the standard PN-EN 1992-1-1 Eurokod 2 [38], chloride ion concentration with a critical value of $C_{K}$ = 0.4% of cement mass. The calculations of chloride concentration changes at the reinforcement–concrete interface of the lagging (x = 15, x = 25, x = 35, and x = 40 mm), which were ascertained according to the known solution of the diffusion Equation (2), assuming the $D^{1}$ value of the diffusion coefficient for the calculations, determined using a thermodynamic migration model. At the edge of the concrete, the chloride concentration was assumed to equal $C_{0}$ = 0.8% of cement mass. Computer-determined changes in time *t* of chloride concentration at the reinforcement–concrete interface of the lagging (i.e., 15, 25, 35 mm, and 40 mm, respectively) are shown in Figure 9.

The corrosion hazard of the reinforcement in B1 concrete will occur after just 2 years of using the structure with the applied lagging thickness of 15 mm, while with the application of a lagging thickness of 25 mm the corrosion hazard will occur after 5 years. Application of the minimum thickness of lagging around the reinforcement required by the standard EN-PN 1992-1-1 [38] in construction class S2 and exposure class XD2/D3 (amounting to 35 and 40 mm, respectively) would avoid corrosion risks to the reinforcement for a period of 10 and 12 years, respectively, at the lagging thicknesses considered. The corrosion hazard of reinforcement in B2 concrete will occur after just 7 years of operation in structures with the applied lagging thickness of 15 mm; by contrast, with the application of lagging thickness of 25 mm, the corrosion hazard will occur after 20 years. Application of the minimum thickness of lagging of reinforcement required by the standard EN-PN 1992-1-1 [38] for construction class S2 and exposure class XD2/D3 (amounting to 35 and 40 mm, respectively) would avoid the corrosion risk of reinforcement for a period of 38 and 49 years, respectively, at the lagging thicknesses considered. The corrosion hazard of reinforcement in B3 concrete will occur already after 6 years of operation in structures with the applied lagging thickness of 15 mm; by contrast, with the application of lagging thickness of 25 mm, the corrosion hazard will occur after 15 years. Application of the minimum thickness of lagging of reinforcement required by the standard EN-PN 1992-1-1 for construction class S2 and exposure class XD2/D3 (amounting to 35 and 40 mm, respectively) would avoid the corrosion risk of reinforcement for a period of 30 and 39 years, respectively, at the lagging thicknesses considered.

### 4.6. Determination of Corrosion Rate of Reinforcing Steel Using the Linear Polarization Method (LPR)

**Figure 10:**
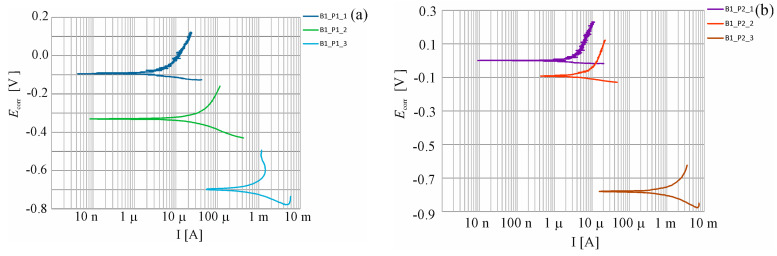

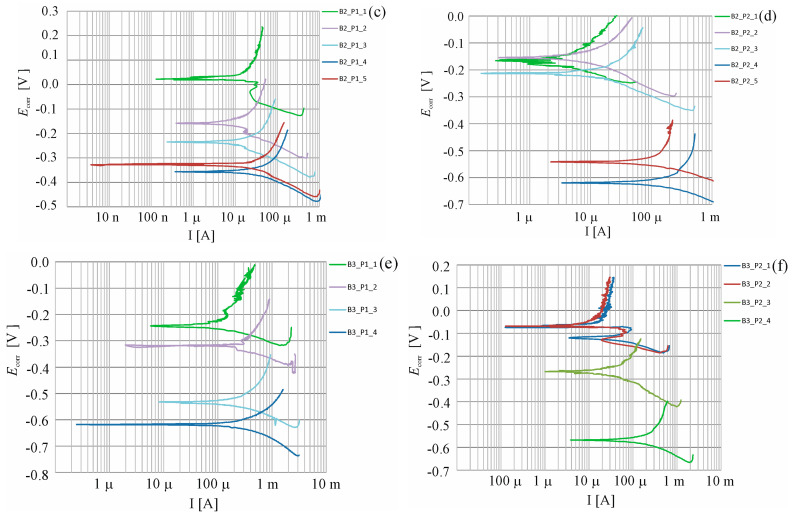
Potentiodynamic polarization curves for steel reinforcement in concrete obtained for the selected specimens: (**a**) B1_P1; (**b**) B1_P2; (**c**) B2_P1; (**d**) B2_P2; (**e**) B3_P1; and (**f**) B3_P2, after successive cycles of chloride ion migration to concrete.

The specimens, for which a number of measurements were taken during the whole research process, were analyzed. They were the B1_P1, B1_P2 and B2_P1, B2_P2 and B3_P1, and B3_P2 specimens. The first measurement was a reference, prior to the chloride migration in the concrete. The second measurement was taken after 7 days of chloride migration onto concrete. After waiting a further 7 days, the third measurement was taken after 14 days of chloride migration into concrete. After waiting 7 days again, the fourth measurement was taken, 21 days after chloride migration into the concrete. Likewise, the fifth measurement, taken after 28 days of chloride migration into the concrete and after waiting 7 days. Three seven-day cycles of chloride ion recharge with an electric field were performed for B1 concrete. For B2 concrete, five seven-day cycles of chloride ion recharge were carried out using an electric field. For B3 concrete, four seven-day cycles of recharging chloride ions with an electric field were carried out. Each time the seven-day migration process was completed, post measurements of the corrosion current density were made after waiting seven days from the electric system being turned off. Charging cycles were applied until the corrosion current density value that would indicate advanced corrosion was obtained. Exemplary shapes for the six selected measuring elements are illustrated in Figure 10.

A similar change in the distribution of polarization curves over time was observed for the specimens B1_P1 and B1_P2 (Figure 9a,b) after the first reference measurement of corrosion potential, taken prior to migration $(E_{corr}=–95\left(B1\_P1\;\_1\right);–2\left(B1\_P2\;\_1\right)$ mV. Then, in both cases, after the first migratory recharge, a decrease in the corrosion potentials were observed, with values: Δ*E_corr_* = 236 mV $\left(B1\_P1\;\_2\right)$, and Δ*E_corr_* = 90 mV $\left(B1\_P2\;\_2\right)$, which can indicate the beginning of the corrosion process. After another recharge, corrosion potential values were observed in both probes, indicating a very high probability of corrosion $(E_{corr}=–694\left(B1\_P1\;\_3\right);–781\left(B1\_P1\;\_3\right)$ mV. In B2 concrete, a decrease in corrosion potential value was observed, from the initial value of $(E_{corr}=–141\left(B2\_P1\;\_1\right);–170\left(B2\_P1\;\_2\right)$ mV to the value at the last measurement of $(E_{corr}=–328\left(B2\_P1\;\_5\right);–429\left(B2\_P2\;\_5\right)$ mV. In sample B2_P2, there was a slight increase in the corrosion potential by a value of Δ*E_corr_* = 15 mV. After the second charge, the values of the corrosion potential in both samples reached a value above the 200 mV $(E_{corr}=–237\left(B2\_P1\;\_2\right);–214\left(B2\_P2\;\_2\right)$ mV, which may indicate a 5% corrosion potential. After the third charge, corrosion potential values obtained in both samples were of the value $(E_{corr}=–357\left(B2\_P1\;\_3\right);–614\left(B2\_P2\;\_3\right)$ mV, indicating a high probability of corrosion.

In B3 concrete, a decrease in corrosion potential was also observed during all test cycles, but the initial values in one of the samples $(E_{corr}=–242\left(B3\_P1\;\_1\right);–320\left(B3\_P1\;\_2\right)$ mV could already indicate a 50% possibility of corrosion. However, in sample B3_P2, in the first two measurements, the value of corrosion potential $\;(E_{corr}=–23\left(B3\_P1\;\_1\right);–49\left(B3\_P1\;\_2\right)$ mV indicated the absence of corrosion. After a further two recharges, corrosion potential values obtained in both samples $(E_{corr}=–618\left(B3\_P1\;\_1\right);–568\left(B3\_P1\;\_2\right)$ mV were indicative of a 95% possibility of corrosion occurrence [39].

**Figure 11:**
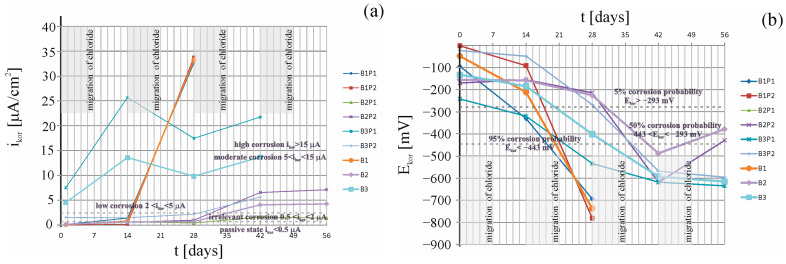
Distributions of (**a**) the corrosion current densities, and (**b**) the corrosion potential, obtained for selected specimens B1P1, B1P2, B2P1, B2P2, B3P1, and B3P2: 1₋before chloride migration; 2₋after 7 days, 3₋after 14 days, 4₋after 21, and 5₋after 28 days of migration.

Figure 11a presents a comparison of the results from six measurements of corrosion current density (*i_corr_*) of the steel reinforcement in concrete from two chosen test elements made of tested concretes. Figure 11b shows a comparison of results from six measurements of corrosion potential (*E_corr_*) of the steel reinforcement in concrete from two chosen test elements made of tested concretes.

The analysis was made on the basis of assumptions made in the work [40]. The first reference measurement taken prior to migration indicated that the average value of corrosion current intensity (B1 (${\bar{i}}_{corr}=$ 0.1 µA); B2 (${\bar{i}}_{corr}=$ 0.18 µA)) suggested the irrelevant corrosion in B1 and B2 concretes. In contrast, concrete B3 (${\bar{i}}_{corr}=$ 4.52 µA) suggested a low corrosion. Another measurement taken boht after 7 days of chloride ions migration under the accelerated action of the electric field and 7 days after switching off the system indicated that the increase in the average corrosion current rate was fastest (an 88% increase) in B1 concrete (${\bar{\Delta i}}_{corr}=$ 0.7 µA), which was also followed by a large increase of 72% in B2 concrete (${\bar{\Delta i}}_{corr}=$ 0.45 µA), and a marginally smaller one (a 67% increase) in B3 concrete (${\bar{\Delta i}}_{corr}=$ 9.06 µA). After another 7-day charging with chloride ions, a massive increase was found for concrete B1 (${\bar{\Delta i}}_{corr}$ = 32.37 µA), reaching the value of high corrosion (${\bar{i}}_{corr}=$ 32.37 µA). On the other hand, in B2 concrete we saw a slight (11%) increase (${\bar{\Delta i}}_{corr}$ = 0.08 µA), and in B2 concrete there was even a decrease of 38%. Due to the high values of corrosion current in concrete B1, measurements of corrosion current velocity were terminated after the second recharge. In the other two concretes, another 7-day recharge of chloride ions was applied. After this third migration cycle, the measured value of the average corrosion current in concrete B2 showed an increase of ${\bar{\Delta i}}_{corr}$ = 3.37 µA, yielding an average value of ${\bar{i}}_{corr}=$ 4.08 µA, which indicated low corrosion levels. In contrast, the B3 concrete showed an increase in the average corrosion current value of ${\bar{\Delta i}}_{corr}$ = 3.86 µA. The obtained average value of the corrosion current rate was ${\bar{i}}_{corr}=$ 13.68 µA, which indicated very high corrosion. Thus, the recharge process was stopped. The value of chloride ion concentration at the surface of the reinforcing steel far exceeded the critical value of $C_{K}$ = 0.4% for the norm [38]. In B2 concrete, another stage of chloride ion migration was applied, after which the average value ${\bar{i}}_{corr}=$ 4.26 µA was obtained, indicating low corrosion. However, due to the fact that the B2P2 sample yielded a value of ${\bar{i}}_{corr}=$ 7.12 µA, it ultimately indicated high corrosion. However, in sample B2P1, the increase in corrosion rate values was higher than 20 times, so the measurements were stopped.

### 4.7. Analysis of EIS Spectra for Steel in Concrete

**Figure 12:**
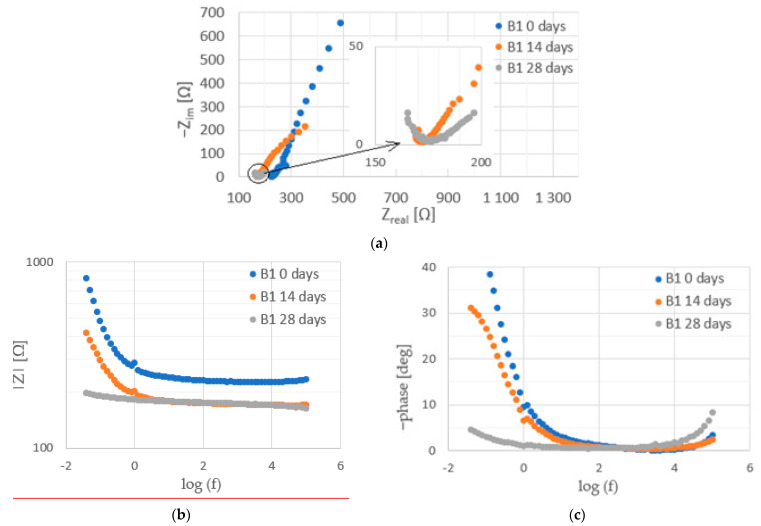
Examples of impedance spectra ((**a**) Nyquist plot, (**b**) impedance, and (**c**) phase angle shift) for steel in B1 concrete during tests.

**Figure 13:**
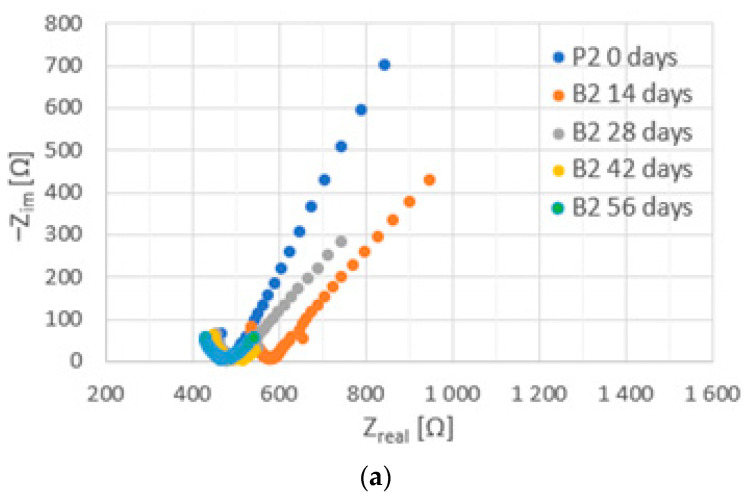

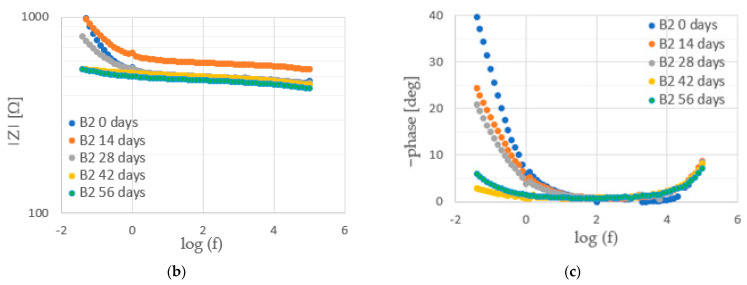
Examples of impedance spectra ((**a**) Nyquist plot, (**b**) impedance, and (**c**) phase angle shift) for steel in B2 concrete during tests.

**Figure 14:**
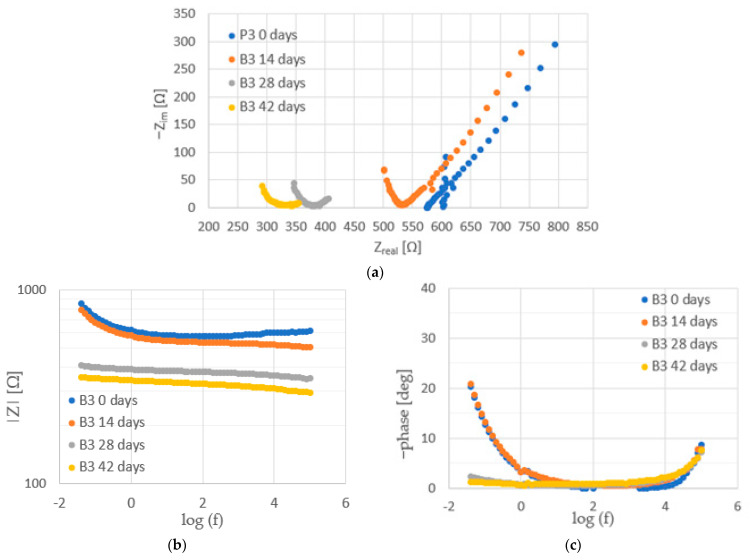
Examples of impedance spectra ((**a**) Nyquist plot, (**b**) impedance, and (**c**) phase angle shift) for steel in B3 concrete during tests.

Before the injection of chloride ions into the concrete, fragments of large arcs were visible on the Nyquist spectra for steel (Figure 12, Figure 13 and Figure 14). This is a characteristic course of the impedance spectra for steels in the passive state. On the Bode diagrams, high impedance values at low frequencies were observed, indicating a low corrosion rate of the steel.

**Figure 15:**
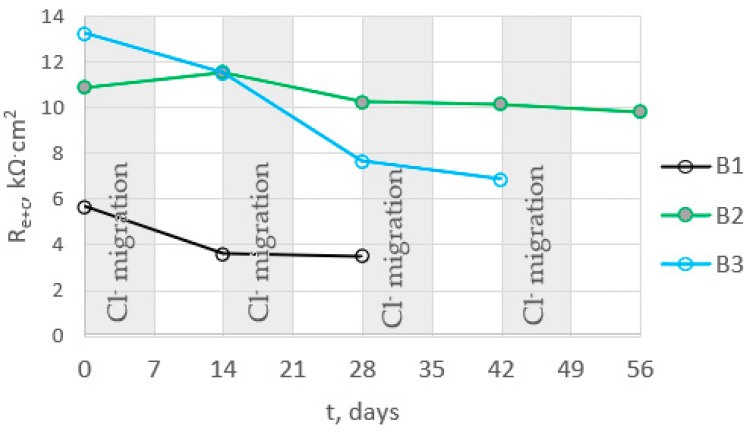
The dependence of the mean values of the sum of the resistance of electrolyte and concrete for B1, B2, and B3 concrete on the migration time of chlorides.

The introduction of chlorides into the concrete resulted in a decrease in the impedance values at high frequencies (corresponding to the sum of the electrolyte and concrete resistance R_e_ + R_c_) associated with an increase in the conductivity of the concrete due to an increase in the ion concentration in the concrete pore liquid. The sum of R_e_ + R_c_ determined from the analysis of the spectra for concretes B2 and B3 was greater than it was for concrete B1 (Figure 15, Table A7, Table A8 and Table A9 in Appendix B), indicating a tighter concrete cover for concretes B2 and B3. This is consistent with the results of the concrete porosity tests (concretes B2 and B3 had lower porosity than B1).

Successive cycles of chloride migration into the concrete resulted in changes in the course of the impedance spectra; a shift in the phase shift angle towards lower frequencies, as well as a decrease in the impedance and phase values at low frequencies, were observed, indicating the development of corrosion processes in the steel. It can be seen that, before the introduction of chlorides, the impedance spectra for steel in B3 concrete were characterized by lower values of impedance and phase shift angle. It can therefore be concluded that the steel in B3 concrete was not as highly corrosion resistant as the steel in B1 and B2 concrete. This may indicate an adverse effect of the use of the admixture on the corrosion of the reinforcement. After two chloride injection cycles, a significant acceleration of corrosion processes is evident.

**Figure 16:**
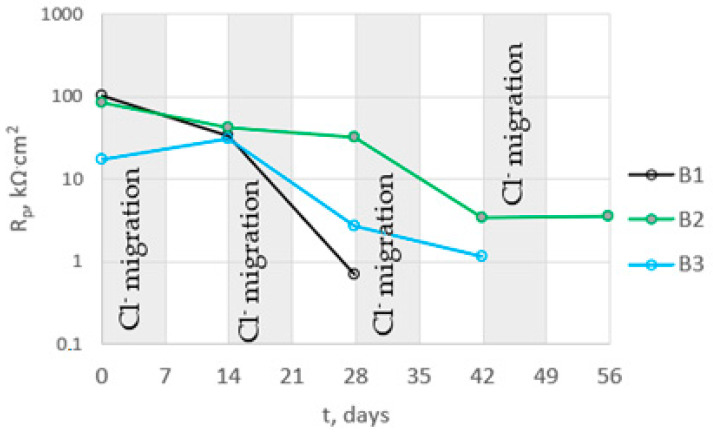
Dependence of mean polarization resistance values for steel in B1, B2, and B3 concrete on chloride migration time.

The determined polarization resistance R_p_ (R_p_ = Rpit + Rct) decreased with the duration of the tests (Figure 16). The value of Rp was inversely proportional to the corrosion rate, so the observed changes in this parameter indicated an increase in the corrosion rate following the introduction of chlorides into the concrete. The rate of change in Rp depended on the type of concrete. The earliest large decrease in Rp, indicating a significant acceleration of steel corrosion, was recorded for B1 concrete. In this concrete, the steel started to corrode intensively after two cycles of chloride migration (2 × 7 days). The Rp value for steel in B3 concrete also decreased significantly after two cycles of chloride migration, but was on average four times higher than the value for B1 concrete, indicating slower corrosion of steel in B3 concrete. The slowest decrease in Rp value was observed for steel in B2 concrete. After two cycles of chloride migration, the values of this parameter were still high, and indicated passive state of the steel. The Rp value for steel in B3 concrete also decreased significantly after two cycles of chloride migration, but was on average four times higher than the value for B1 concrete, indicating slower corrosion of steel in B3 concrete. The slowest decrease in R_p_ value was observed for steel in B2 concrete; after two cycles of chloride migration, the values of this parameter were still high and indicated passivation of the steel. However, after three cycles of chloride migration, the R_p_ values for the steel in B3 concrete decreased significantly, indicating a significant corrosion rate of the steel. The results obtained were consistent with the determined diffusion coefficients of the concrete. The steel in B1 concrete (i.e., in the concrete with the highest diffusion coefficient) started to corrode after the shortest time following chloride introduction into the concrete. The R_p_ values determined from the analysis of the impedance spectra of steel in concrete confirmed the results obtained from the analysis of the steel polarization curves.

**Figure 17:**
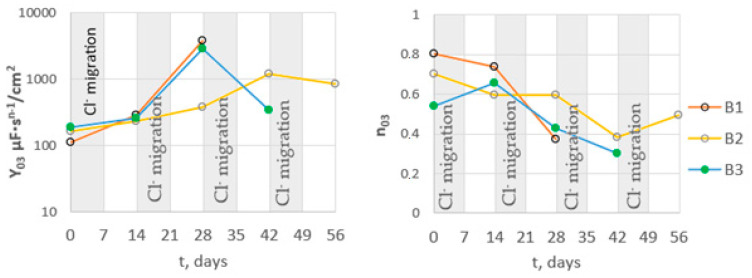
Dependence of the mean values of the parameters of constant phase element describing the double layer on steel in B1, B2, and B3 concrete on the migration time of chlorides.

With increasing chloride migration time, changes in the parameters describing the constant phase element (CPE_dl_) were also observed (Figure 17). The value of the Y_03_ parameter increased with the increase in chloride migration time. This indicated an increase in the capacity of the double layer, associated with an increase in thickness. An increase in parameter Y_03_ was accompanied by a decrease in parameter n. This indicated a deterioration in the homogeneity of the double layer on the steel. Such changes are observed due to a deterioration of the protective properties of the passive layer on the steel and the development of pits on the steel surface.

### 4.8. Results of Phase Identification and Quantitative Analysis of Corrosion Products on Reinforcing Steel, using the Rietveld Method 

**Figure 18:**
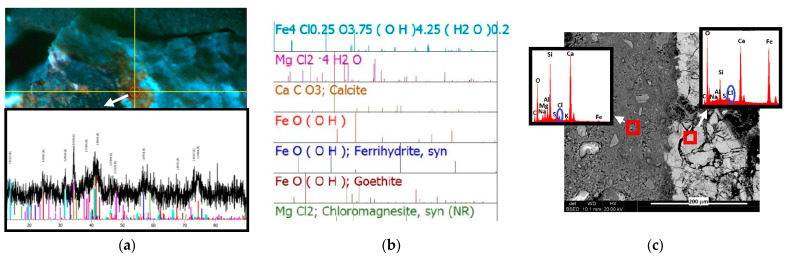
Surface of concrete adjacent to the surface of the bar: (**a**) image from the camera in the X-ray diffractometer showing the area examined using X-ray diffraction (XRD, highlighted in red, with a diameter of 400 μm) and the measured diffractogram at bottom left; (**b**) phase identification made on the basis of the measured diffractogram, which is also included; (**c**) SEM micrographs under backscattered electron (BSE) light and the results of chemical composition analysis from the areas marked on the micrographs (X-ray emission spectra (EDS)).

Scanning electron microscopy (SEM), equipped with microarea chemical analysis capabilities and X-ray microdiffraction (μXRD), were used to study microstructural changes, in addition to changes in the phase composition of corrosion products occurring in the steel–concrete contact zone. These changes were observed during the development of corrosion processes caused by an increase in the concentration of chloride ions in the concrete. Examples of tests performed using X-ray microdiffraction (XRD) and surface micrography are shown in Figure 18a–c.

The use of phase identification showed that there are differences in the iron compounds formed depending on the type of sample. Lepidocrocite and akaganeite are present in all areas tested, while the presence of δ-type iron oxide was only confirmed in the sample from B2 concrete; likewise, goethite was identified only in the sample from B3 concrete. The only iron compound containing chlorine is akaganeite, though chlorine–calcium compounds also appeared in the sample from concrete B3. In addition, the presence of hydrated iron–potassium oxide was possible. No reflections of chlorine, potassium, or sodium compounds were registered in any of the samples.

Table 5 shows the results of quantitative phase analysis of corrosion products.

Only a small amount of bassanite was identified in the B1 concrete sample. Aqueous conversion of bassanite (Ca-SO4 0.5H_2_O) to gypsum (CaSO_4_ 2H_2_O) controls the setting process of gypsum plaster [41], hence it can be assumed that there is a danger of gypsum nucleation in this concrete.

Quartz was not included in the quantitative calculations. In the B1 concrete sample, the proportions of iron compounds were at a similar level; magnetite and lepidocrocite in the amount of about 35% of the mass and akaganeite, i.e., about 29% of the mass. In the sample from concrete B2, the dominant component was iron oxyhydroxide of the type δ, comprising 59% of the mass; the share of lepidocrocite and magnetite was again in similar amounts (10% of the mass and 7% of the mass, respectively), while akaganeite was 24% of the mass. In the B3 concrete sample, goethite was the most abundant at 48.7% of the mass; lepidocrocite was at the same level as it was in the B1 concrete sample, i.e., 35.9% of the mass. In this sample, the share of akaganeite was the lowest, at about 10% of the mass. This was also the lowest share of this mineral in all the samples tested.

It can be concluded that a common feature of the tested samples was the presence of lepidocrocite and akaganeite, although their proportions varied from sample to sample. In each of the samples, a different phase was dominant; in the B1 concrete sample, all three iron compounds were found in similar amounts, while in the B2 concrete sample, half of the tested material was formed of iron oxide type δ, and in sample No. 3, the main phase was goethite. Since lepidocrocite was the result of hydration of goethite (and there was the least amount of it in the B2 concrete sample), it can be assumed that corrosion processes in the B2 concrete sample occurred more slowly than in the other samples.

## 5. Conclusions

The type of cement influences the rate of chloride diffusion in the concrete, and thus the duration of corrosion initiation of the reinforcement by chlorides penetrating into the concrete from the environment.

Of the concretes tested, concrete B2 with CEM III cement was the most resistant to chloride ingress, and therefore provided the longest period of protection for the reinforcement against chloride-initiated corrosion. The fastest, occurring after only two 7-day cycles of chloride migration in the electric field, was the steel corrosion that started in concrete with CEM I cement (concrete B1).

The results of the concrete diffusion and porosity tests correlated with the time of the onset of steel corrosion in the tested concretes due to chloride penetration into the concrete.

Similarly, studies of both the microstructure and changes in the phase composition of corrosion products occurring in the steel –concrete contact zone confirmed these observations as having the smallest amount of lepidocrocite, which was the result of goethite hydration in the smallest amount, occurring in the sample made of B2 concrete.

The additional use of a sealing admixture did not seem to be the most effective strategy, as it can cause a local increase in the volume of pores in the concrete, and, at the same time, a local weakening of the concrete structure, as can be seen from the distribution of the number of pores in a given volume found using computed tomography. According to the study conducted with this method, B2 concrete shows the most uniform distribution of pores of different volumes, with the lowest total number of pores.

The test results obtained were consistent with the results obtained in the work [42], where the use of metallurgical cement in the samples (together with the simultaneous addition of an air-entraining agent) showed better protective properties, as determined using the galvanostatic pulse method.

## Figures and Tables

**Table 1:** Composition, properties, and compressive strength of concrete mixtures.

Constituent	B	B2	B3
Cement	368		
Aggregate	1915		
Water	147		
w/c	0.4		
Plasticizer (0.5% m.c.)			
Sealing admixture (0.8% m. c.)			
Compressive strength f_cm_ MPa	62.4	63.9	56.9
Volume weight $\gamma_{b}$ kg/m^3^	2271	2241	2269

**Table 2:** Depth of penetration of pressurized water.

Concrete	B1.1	B1.2	B1.3	B2.1	B2.2	B2.3	B3.1	B3.2	B3.3
Depth of penetration [mm]	23	20	25	14	6	14	18	18	22
Average depth [mm]	23			11			19		

**Table 3:** Values of the open porosity and mass of a sample made of concrete B1, B2, and B3, determined using the gravimetric method with a hydrostatic balance.

Concrete	Dry Mass g	Water Saturation Mass g	Floating Mass g	Open Porosity %
B1	216.85	225.3	131.4	9
B2	121.36	126.2	72.8	9
B3	148.37	153.4	87.9	8

**Table 4:** Diffusion and migration coefficient values determined using different methods [8].

Mix ID	Diffusion Coefficient Calculated (10^−12^ m^2^/s)							
	$D_{N-E}\;$ $\left(\sigma \right)\;**$	$D_{T}$ $\left(\partial \right)\;***$	$D_{migr}^{1}10^{-3}$ $(s\left(x\right))\;*$	$D_{dyf}^{1}$ $(s\left(x\right))\;*$	D; $(s\left(x\right))\;*$	$D^{t1};\;$ $\left(s\left(x\right)\right)\;*$	$D^{t2}\;$ $\left(s\left(x\right)\right)\;*$	$D^{\;1}$
B1	3.67; (0.28)	0.48; (±0.06)	12.5; (0.69)	12.5; (0.69)	1.20; (0.85)	4.84; (0.34)	2.42; (0.61)	4.84
B2	1.34; (0.05)	2.0; (±0.06)	16; (0.74)	16; (0.74)	2.96; (0.21)	3.84; (0.25)	1.92; (0.62)	1.48
B3	1.86; (0.05)	1.41; (±0.06)	130; (0.48)	130; (0.48)	2.32; (0.67)	5.88; (0.54)	4.52; (0.33)	2.27

* (s(x))—the value of mean squared error in brackets; ** (σ)—the value of standard deviation in brackets; and *** (∂)—the value of error due to the length of the scale in brackets.

**Table 5:** Results of phase analysis of corrosion products.

Identified Phase Components Share [mass %]	B1	B2	B3
Magnetite Fe_3_O_4_	34.7 ± 2.1	-	-
Lepidocrocite γ-FeO(OH)	36.2 ± 1.8	10.0 ± 1.1	35.9 ± 1.7
Akaganeite Fe_4_Cl_0.44_O_3.55_(OH)_4.44_ (H_2_O)_0.2_	28.7 ± 2.0	24.0 ± 1.7	10.9 ± 0.7
Bassanite CaSO_4_(H _2_ O)_0.5_	<1.0	-	-
Feroxyhyte δ-FeOOH	-	59.0 ± 2.1	-
Goethite α-FeO(OH)	-	-	48.7 ± 2.0
CaCl_2_	-	-	3.3 ± 0.8

## Data Availability

The data presented in this study are available on request from the corresponding author.

## Appendix A

**Table A1:** Compressive strength results of B1 concrete after 1, 2, 7 and 28 days of curing.

Test Date [Day]	Compressive Force [kN]	Compressive Strength [MPa]	Average Compressive Strength [Mpa]
1	456.3	20.3	20.3
	441.7	19.6	
	470.2	20.9	
2	834.7	37.1	38.6
	896.54	39.8	
	875.23	38.9	
7	1303.7	57,9	59.1
	1328.2	59,0	
	1356.4	60,3	
28	1452.2	64.5	62.4
	1422.7	63.2	
	1339.5	59.5	

**Table A2:** Compressive strength results of B2 concrete after 1, 2, 7 and 28 days of curing.

Test Date [Day]	Compressive Force [kN]	Compressive Strength [Mpa]	Average Compressive Strength [Mpa]
1	384.0	17.1	17.8
	397.4	17.7	
	418.2	18.6	
2	713.0	31.7	31.4
	719.4	32.0	
	683.9	30.4	
7	1008.3	44.8	46.0
	1027.5	45.7	
	1069.0	47.5	
28	1413.7	62.8	63.9
	1454.0	64.6	
	1445.3	64.2	

**Table A3:** Compressive strength results of B3 concrete after 1, 2, 7 and 28 days of curing.

Test Date [Day]	Compressive Force [kN]	Compressive Strength [Mpa]	Average Compressive Strength [Mpa]
1	335.8	14.9	14.7
	327.1	14.5	
	330.7	14.7	
2	523.7	23.3	38.6
	514.6	22.9	
	522.0	23.2	
7	861.7	38.3	59.1
	983.2	43.7	
	861.3	38.3	
28	1228.5	54.6	62.4
	1304.7	58.0	
	1309.4	58.2	

## Appendix B

**Table A4:** Comparison of results from analyzing polarization curves obtained for two selected specimens (B1.1 and B1.2), measuring before and after 14, 28, 42 and 56 days of chloride migration.

Measure No.	Time Days	E_corr_ mV	b_a_ mV	b_c_ mV	R_p_ kΩ	R_p_A kΩcm^2^	I_corr_ μA/cm^2^	V_r_ mm/year
B1.1	0	−95	399	25	3.01	68.33	0.15	0.002
B1.2	0	−2	345	12	4.79	108.73	0.05	0.001
B1.1	14	−333	394	72	0.82	18.70	1.41	0.016
B1.2	14	−92	622	30	2.93	66.51	0.19	0.002
B1.1	28	−694	61	710	0,03	0.75	32.56	0.378
B1.2	28	−781	447	49	0.03	0.57	33.79	0.392

**Table A5:** Comparison of results from analyzing polarization curves obtained for two selected specimens (B2.1 and B2.2), measuring before and after 14, 28, 42, and 56 days of chloride migration.

Measure No.	Time Days	E_corr_ mV	b_a_ mV	b_c_ mV	R_p_ kΩ	R_p_A kΩcm^2^	I_corr_ μA/cm^2^	V_r_ mm/year
B2.1	0	−10	701	54	4.57	103.74	0.21	0.002
B2.2	0	−170	239	52	5.71	129.59	0.14	0.002
B2.1	14	−161	500	59	1.52	34.50	0.66	0.008
B2.2	14	−155	265	109	2.52	57.11	0.59	0.007
B2.1	28	−237	480	23	1.01	22.93	0.42	0.005
B2.2	28	−214	440	82	1.33	30.21	0.99	0.012
B2.1	42	−357	78	92	0.51	11.49	1.60	0.019
B2.2	42	−619	85	70	0.11	2.54	6.56	0.076
B2.1	56	−328	96	84	0.61	13.87	1.40	0.016
B2.2	56	−429	368	69	0.16	3.54	7.12	0.083

**Table A6:** Comparison of results from analyzing polarization curves obtained for two selected specimens (B3.1 and B3.2), measuring before and after 14, 28, 42, and 56 days of chloride migration.

Measure No.	Time Days	E_corr_ mV	b_a_ mV	b_c_ mV	R_p_ kΩ	R_p_A kΩcm^2^	I_corr_ μA/cm^2^	V_r_ mm/Year
B3.1	0	−242	172	94	0.16	3.52	7.50	0.087
B3.2	0	−23	200	119	0.93	21.11	1.53	0.018
B3.1	14	−320	210	127	0.06	1.34	25.66	0.298
B3.2	14	−49	566	90	1.00	22.70	1.49	0.017
B3.1	28	−534	579	80	0.08	1.75	17.46	0.203
B3.2	28	−268	315	107	0.70	15.89	2.18	0.025
B3.1	42	−618	476	78	0.06	1.34	21.73	0.252
B3.2	42	−568	590	34	0.11	2.47	5.64	0.065

**Table A7:** Parameters of the electrical equivalent circuit elements describing the EIS spectra of steel in B1 concrete.

Sample	t Days	R_e_ + R_c_ kΩ/cm^2^	CPE_c_		CPE_pas_		R_pit_ kΩ/cm^2^	CPE_ct_		R_ct_ kΩ/cm^2^	W µF·s^n−1^/cm^2^
			Y_01_ nF·s^n−1^/cm^2^	n_1_	Y_02_ µF·s^n−1^/cm^2^	n_2_		Y_03_ µF·s^n−1^/cm^2^	n_3_		
B1.1	0	5.2	0.019	1.00	54	0.79	0.71	131	0.77	60.5	0.000
B1.2	0	6.2	0.383	0.82	56	0.80	1.97	91	0.83	146	0.002
B1.1	14	3.7	0.014	1.00	114	0.32	0.31	262	0.76	17.1	0.000
B1.2	14	3.5	0.393	0.80	91	0.53	0.20	312	0.72	49.1	0.221
B1.1	28	3.6	0.120	0.96	80	0.30	0.42	3216	0.43	0.48	0.099
B1.2	28	3.4	0.048	1.00	2	0.73	0.16	1164	0.31	0.33	7040

**Table A8:** Parameters of the electrical equivalent circuit elements describing the EIS spectra of steel in B2 concrete.

Sample	t Days	R_e_ + R_c_ kΩ/cm^2^	CPE_c_		CPE_pas_		R_pit_ kΩ/cm^2^	CPE_ct_		R_ct_ kΩ/cm^2^	W µF·s^n−1^/cm^2^
			Y_01_ nF·s^n−1^/cm^2^	n_1_	Y_02_ µF·s^n−1^/cm^2^	n_2_		Y_03_ µF·s^n−1^/cm^2^	n_3_		
B2.1	0	11.1	0.023	1.00	44	0.49	0.5	198	0.69	84.1	0.001
B2.2	0	10.8	0.082	0.90	25	0.64	0.6	125	0.72	84.2	0.006
B2.1	14	12.2	0.073	0.91	31	0.30	1.4	136	0.63	48.8	1.759
B2.1	28	11.0	0.098	0.88	12	0.40	1.2	335	0.56	34.5	0.060
B2.2	28	10.3	0.087	0.90	34	0.30	1.3	216	0.60	30.6	0.000
B2.2	42	10.2	0.186	0.84	28	0.32	1.4	1189	0.38	2.04	647
B2.2	56	9.8	0.205	0.83	31	0.32	1.2	846	0.50	2.27	0.000

**Table A9:** Parameters of the electrical equivalent circuit elements describing the EIS spectra of steel in B3 concrete.

Sample	t Days	R_e_ + R_c_ kΩ/cm^2^	CPE_c_		CPE_pas_		R_pit_ kΩ/cm^2^	CPE_ct_		R_ct_ kΩ/cm^2^	W µF·s^n−1^/cm^2^
			Y_01_ nF·s^n−1^/cm^2^	n_1_	Y_02_ µF·s^n−1^/cm^2^	n_2_		Y_03_ µF·s^n−1^/cm^2^	n_3_		
B3.1	0	13.3	0.017	1.00	215	0.74	2.6	70	0.55	12.18	56.00
B3.2	0	13.2	0.094	0.86	43	0.70	0.6	313	0.63	18.7	0.000
B3.1	14	11.6	0.049	0.94	17	0.46	0.7	264	0.59	42.15	7.181
B3.2	14	11.5	0.301	0.80	81	0.61	0.9	250	0.73	17.3	0.000
B3.1	28	7.7	0.069	0.92	9	0.42	0.9	2194	0.33	1.15	0.000
B3.2	28	7.6	0.323	0.82	193	0.32	1.1	2043	0.53	2.23	0.097
B3.1	42	6.9	0.243	0.85	0.4	0.81	0.3	346	0.30	0.88	7546
